# Insurance against Surgical Operations

**Published:** 1904-03-19

**Authors:** 


### INSURANCE AGAINST SURGICAL OPERATIONS.

It is a trite observation that modern civilisation, however decided its advantages, has increased greatly the strain and stress of life. The effort for success is encouraged by a more ample reward, and the consequences of failure carry with them a heavier penalty. To stay or halt, means not infrequently the loss of opportunities escaped beyond recall. Hence the struggle for existence or success, as the case may be, keen as it is in the very nature of things, has an added anxiety from the thought that some cruel chance may compel a period of inactivity which in its turn may spell disaster. To every man at times there comes the thought that illness or accident, against which no care or wisdom will avail, may neutralise all his efforts and destroy his most promising schemes; and for most at least there is the sharpened anxiety that the consequences of such a misfortune will not be merely individual but will include the weal of others who share with him a common risk. It is out of this experience that there has grown the prinicple which finds its expression in the habit of insurance against various possible contingencies. Life involves dangers, and the wise man both recognises these and endeavours to provide for them. To argue in favour of such a course nowadays is, however, hardly necessary. "The slings and arrows of outrageous fortune" constitute an appreciable element in the calculations of the prudent, and the recognition of this factor claims its due regard. Commercial enterprise has done much to proclaim and emphasise the necessity for this recognition, and at the same time it has stimulated economic ingenuity to devise methods by which misfortune may in some measure be rendered less disastrous. Hence, insurance companies concerned with various interests have multiplied and flourished. Death, accident, disease, and other risks have been brought within the protected area, and the practice is a growing and extending one. It is impossible to question that much good has attended this development. And this not only in the mitigation of the misfortune when such occurs, but also in the relief of some part of the anxiety and care which a survey of the possibilities of life is calculated to provoke. The latest extension of the insurance habit applies to the risk of surgical operations. Whatever else may be said of modern surgery it has certainly enormously multiplied the number of operations performed. Happily it is true that, thanks to anæsthetics and antiseptics, the risk to life in the great majority of these operations is comparatively slight. Yet the multiplication of the number means that the chances of any individual coming under the surgeon's knife are proportionately increased. The question can be made a matter of actuarial calculation, and thus as a financial risk comes within the scope of a possible insurance policy. Now, to many households the expense of a surgical operation and the incidental charges arising out of it, present a most serious aspect. Even when they can be met they often mean a burden which adds considerably to the anxieties of the situation. There seems, therefore, ample justification for the proposal to bring the risk of such an event into the list of contingencies which may be met by the practice of insurance. From a prospectus now lying before us, we observe that for an annual premium of a few shillings a policy insuring the payment of all expenses (not exceeding £100) incurred in connection with various surgical operations, can be secured. This seems to us an admirable provision against a risk to which all are liable, and that, too, in an increasing degree. No one whose opinion is worth listening to would argue that surgical fees are excessive, but the total charges in connection with a serious surgical operation must needs seem heavy to those of moderate means, more especially as, occurring unexpectedly, no provision is made for the event. The proposal just alluded to meets this difficulty with a scheme which has a sound economic basis. It has another advantage. Many patients, who are most unwilling to accept charitable relief, are undoubtedly compelled to seek admission to a hospital when they are suddenly brought face to face with the necessity for a surgical operation and all that this means. An insurance to provide for the expenses thus unexpectedly arising affords an opportunity for nearly everyone to meet such an emergency when it occurs without an appeal to the charity of others. Useful and sound in itself, the proposal may, therefore, be further commended because it encourages the virtues of thrift, self-reliance, and independence, qualities which not a few of our existing social arrangements tend rather to weaken than to develop.

